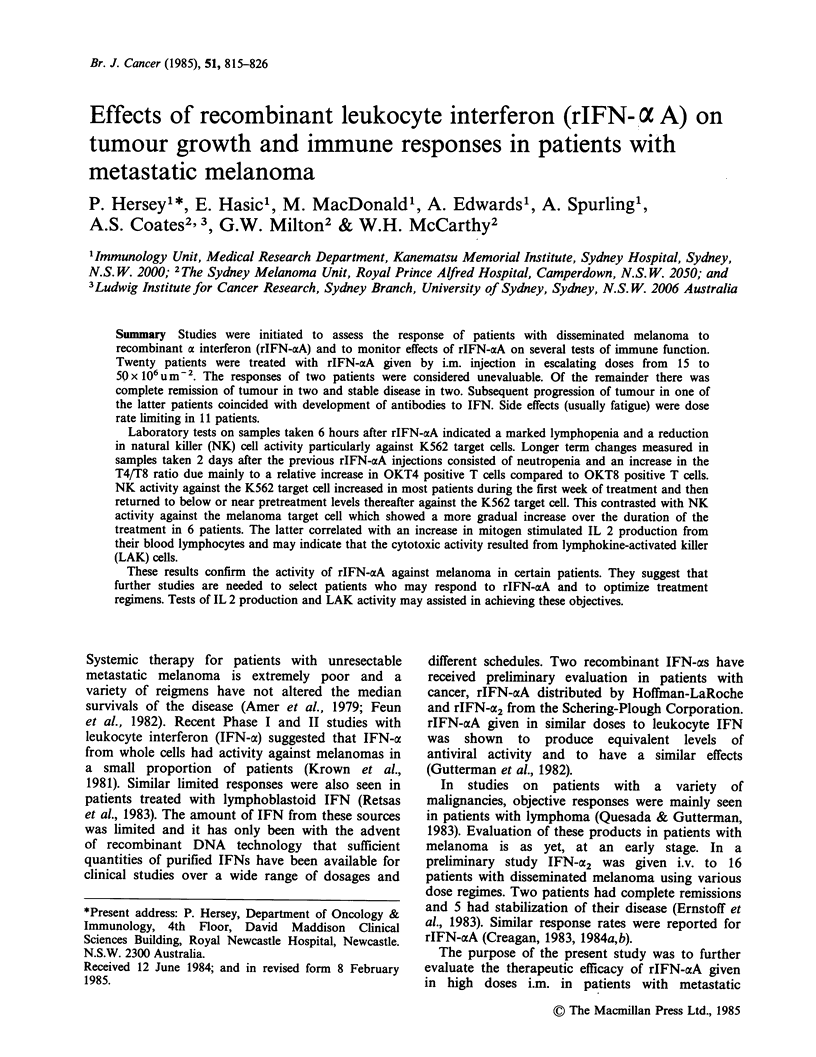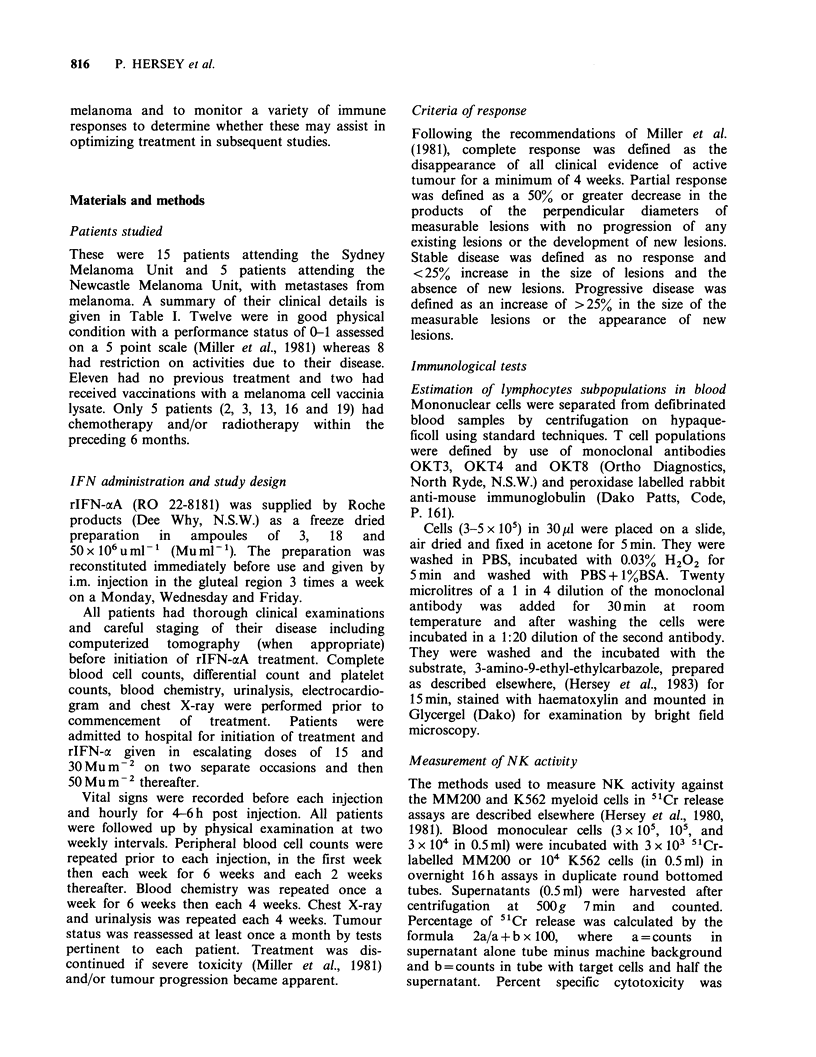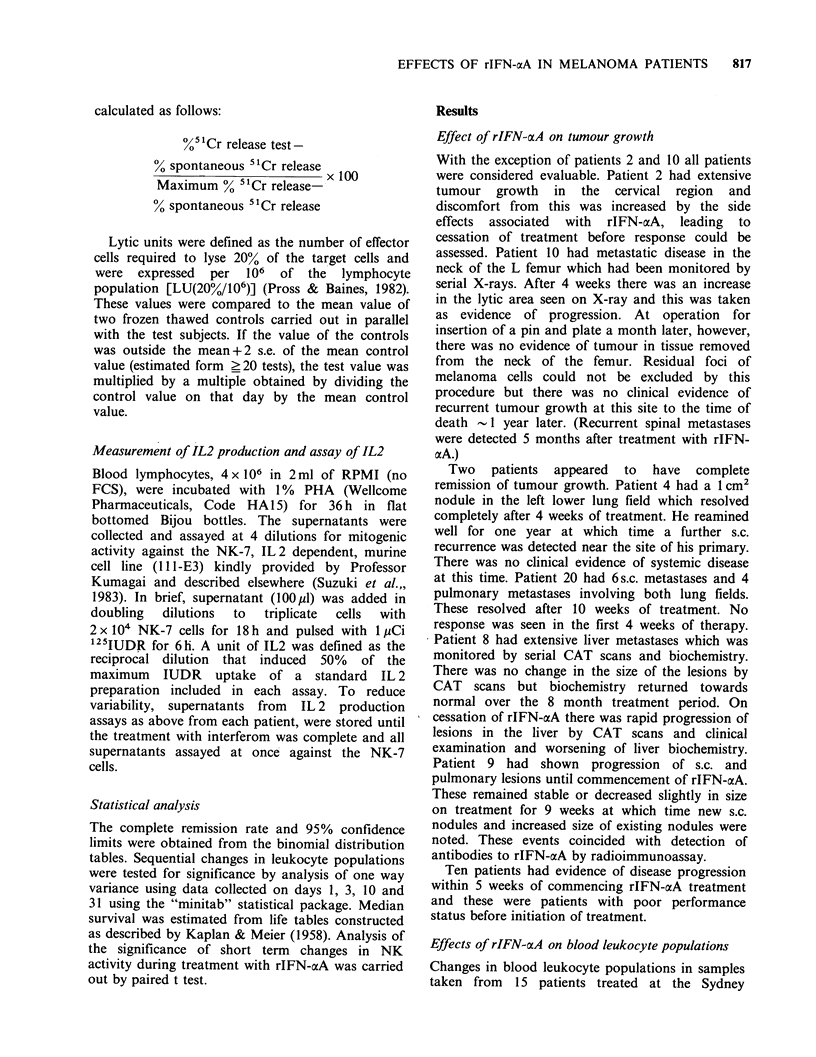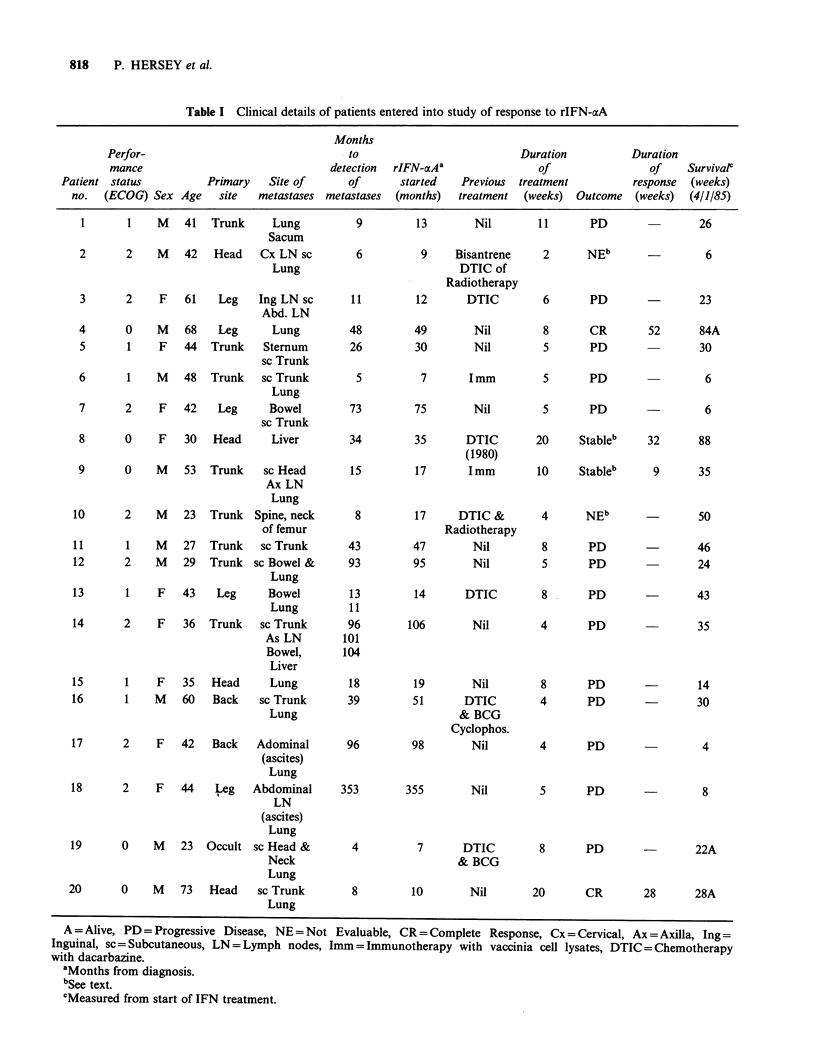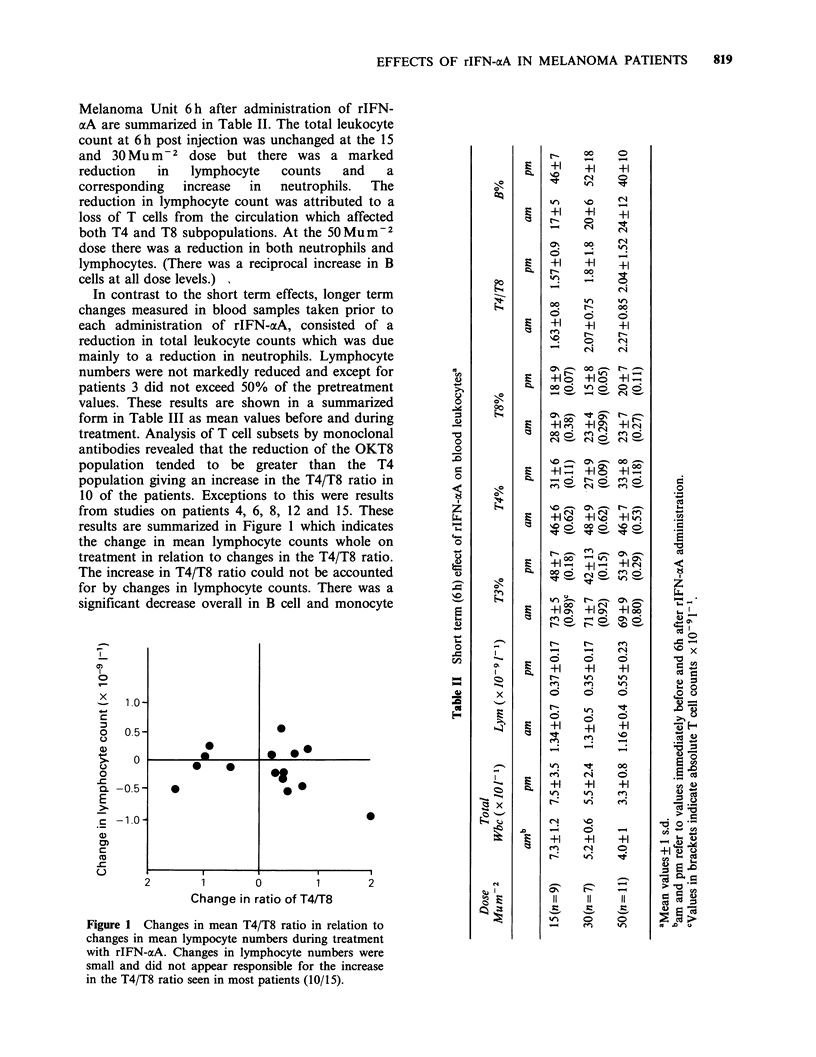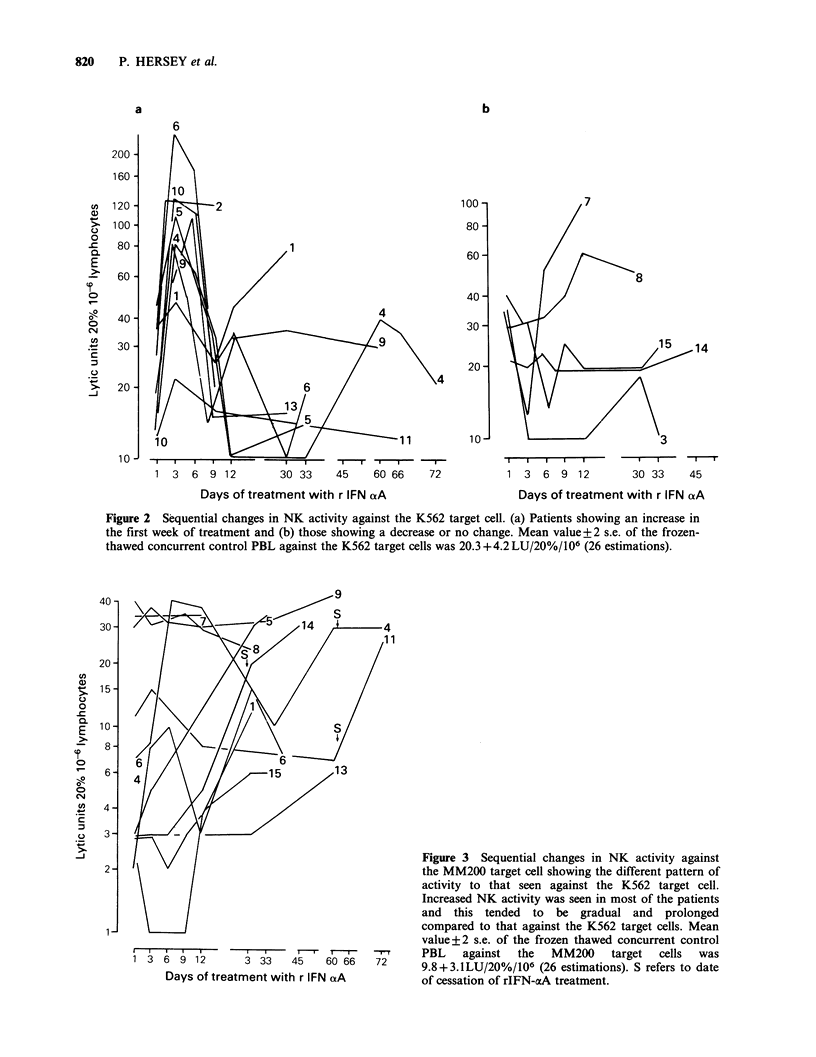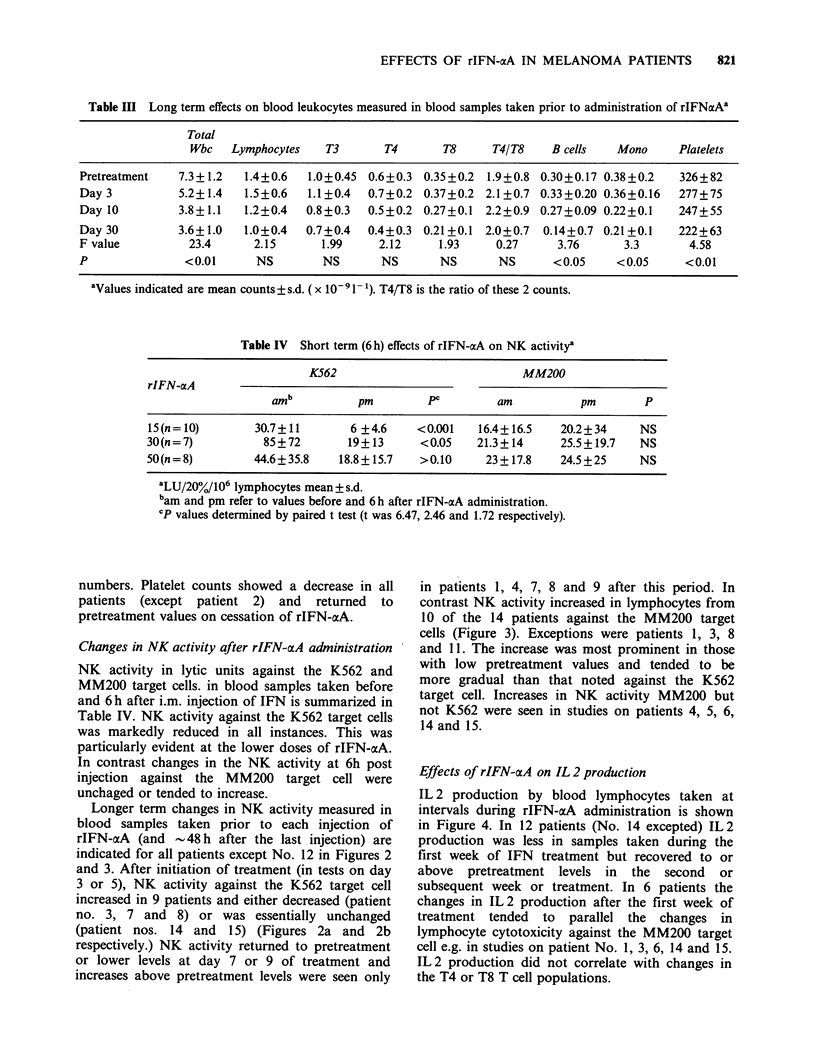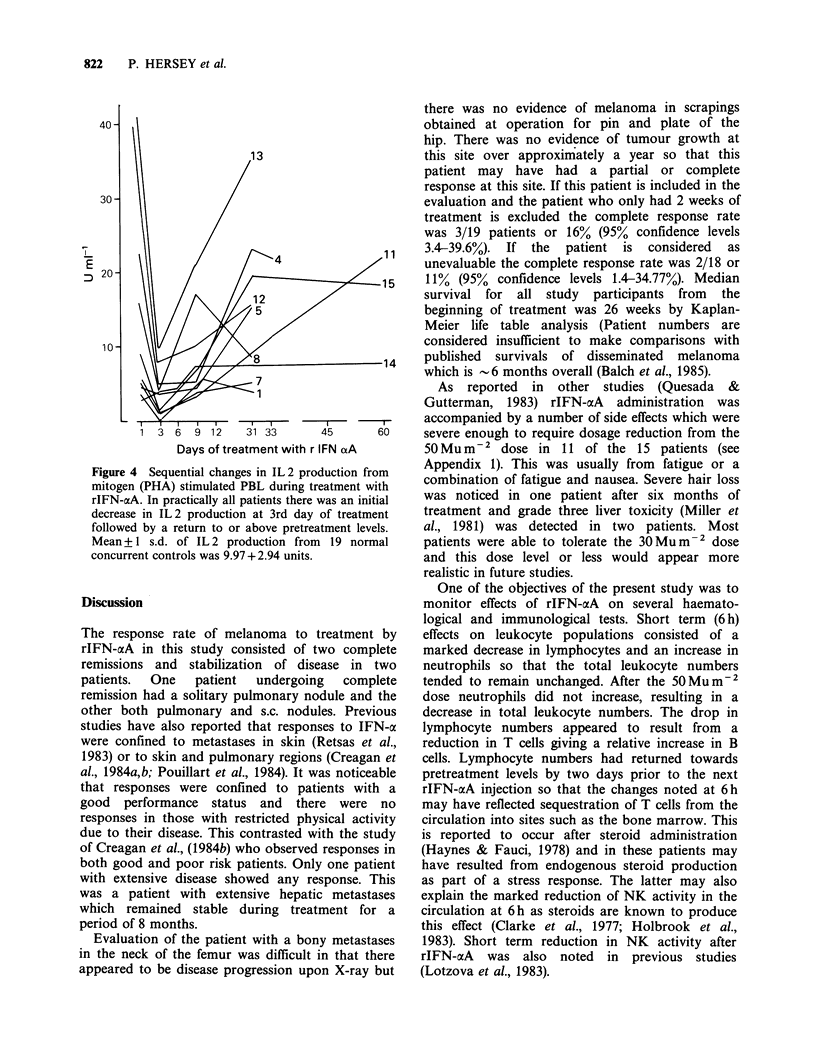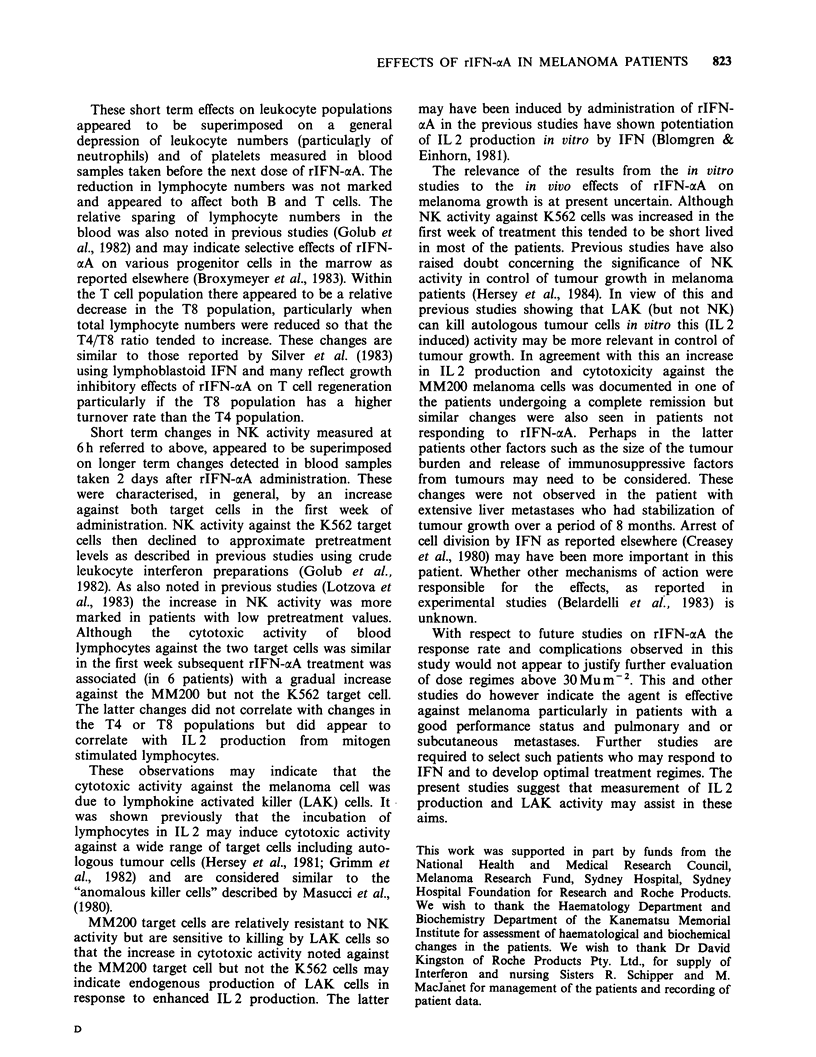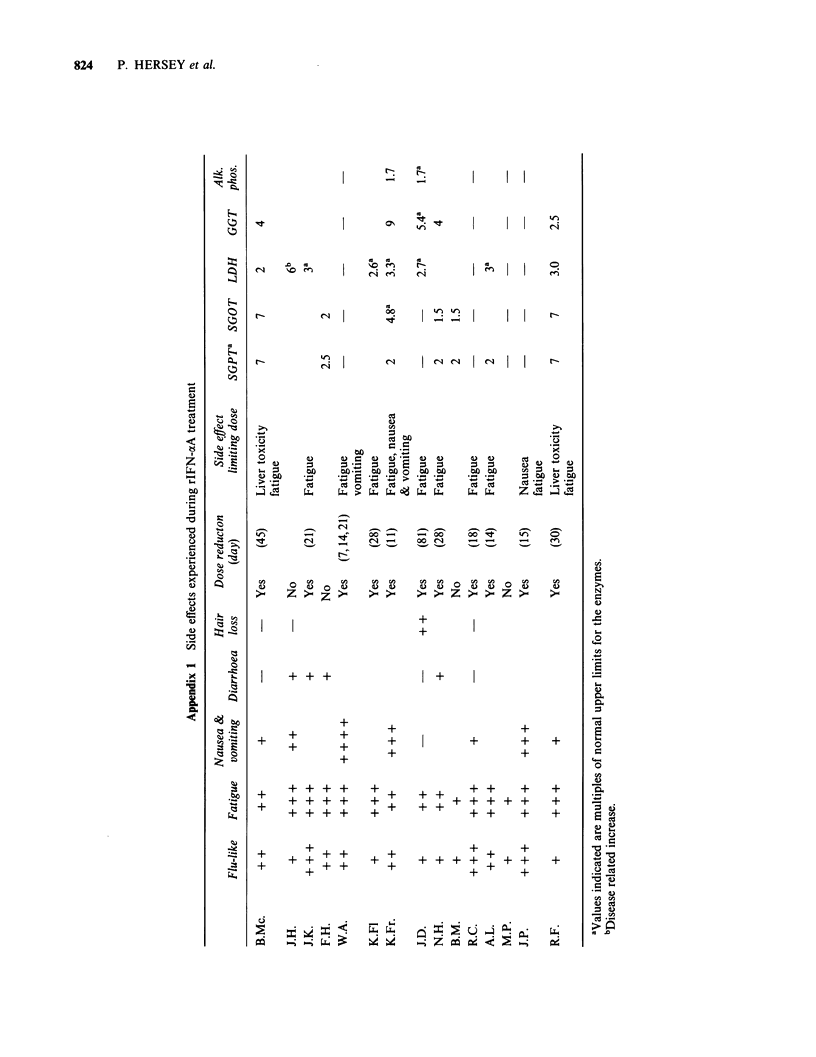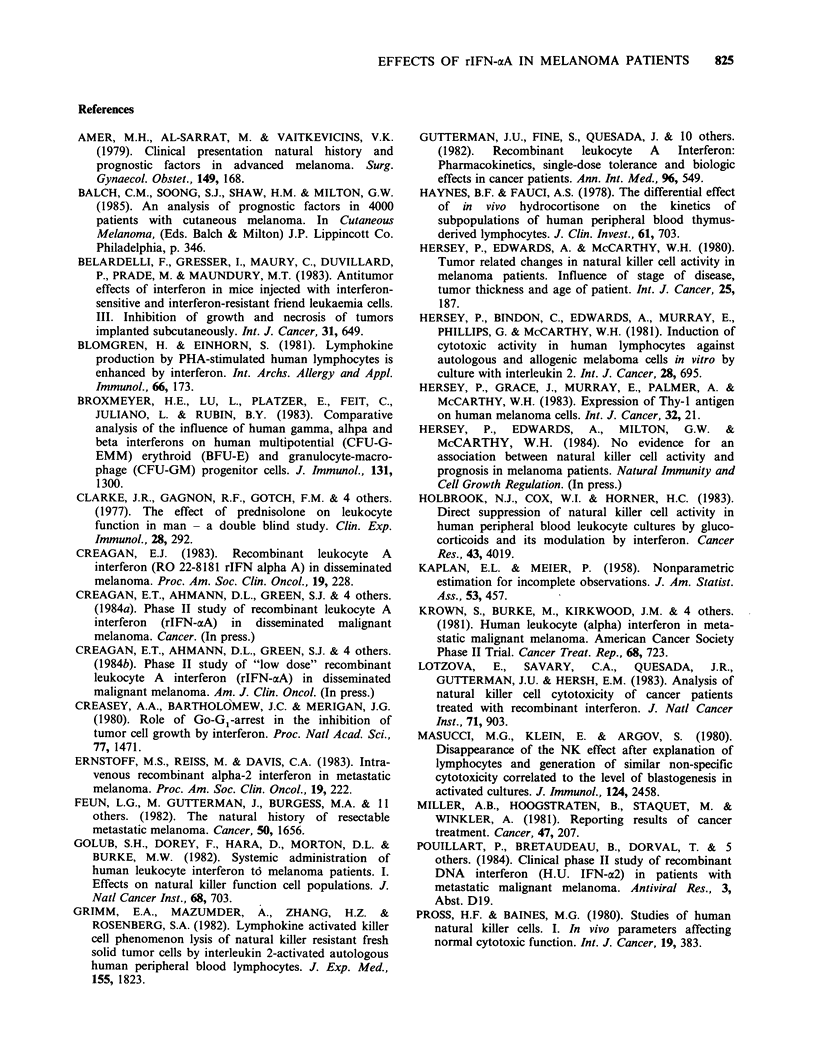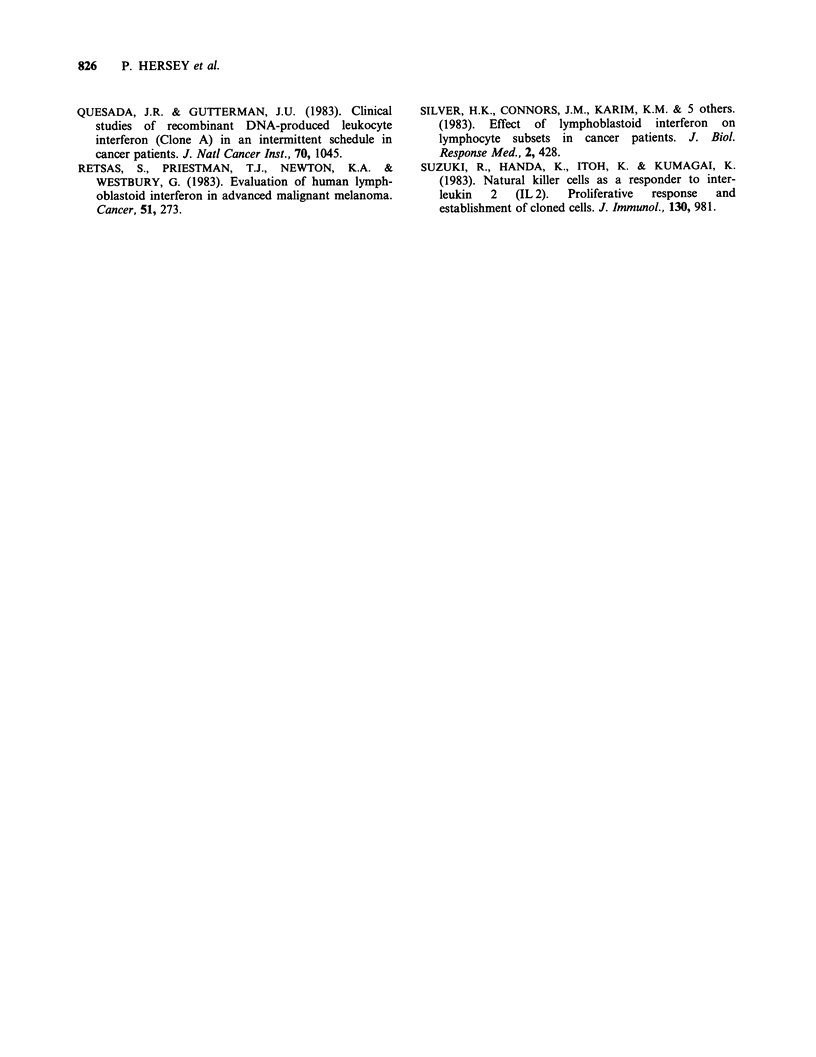# Effects of recombinant leukocyte interferon (rIFN-alpha A) on tumour growth and immune responses in patients with metastatic melanoma.

**DOI:** 10.1038/bjc.1985.127

**Published:** 1985-06

**Authors:** P. Hersey, E. Hasic, M. MacDonald, A. Edwards, A. Spurling, A. S. Coates, G. W. Milton, W. H. McCarthy

## Abstract

Studies were initiated to assess the response of patients with disseminated melanoma to recombinant alpha interferon (rIFN-alpha A) and to monitor effects of rIFN-alpha A on several tests of immune function. Twenty patients were treated with rIFN-alpha A given by i.m. injection in escalating doses from 15 to 50 X 10(6) um-2. The responses of two patients were considered unevaluable. Of the remainder there was complete remission of tumour in two and stable disease in two. Subsequent progression of tumour in one of the latter patients coincided with development of antibodies to IFN. Side effects (usually fatigue) were dose rate limiting in 11 patients. Laboratory tests on samples taken 6 hours after rIFN-alpha A indicated a marked lymphopenia and a reduction in natural killer (NK) cell activity particularly against K562 target cells. Longer term changes measured in samples taken 2 days after the previous rIFN-alpha A injections consisted of neutropenia and an increase in the T4/T8 ratio due mainly to a relative increase in OKT4 positive T cells compared to OKT8 positive T cells. NK activity against the K562 target cell increased in most patients during the first week of treatment and then returned to below or near pretreatment levels thereafter against the K562 target cell. This contrasted with NK activity against the melanoma target cell which showed a more gradual increase over the duration of the treatment in 6 patients. The latter correlated with an increase in mitogen stimulated IL 2 production from their blood lymphocytes and may indicate that the cytotoxic activity resulted from lymphokine-activated killer (LAK) cells. These results confirm the activity of rIFN-alpha A against melanoma in certain patients. They suggest that further studies are needed to select patients who may respond to rIFN-alpha A and to optimize treatment regimens. Tests of IL 2 production and LAK activity may assisted in achieving these objectives.


*Br. J. Cancer* (1985), **51**, 815–826

### Effects of recombinant leukocyte interferon (rIFN-α A) on tumour growth and immune responses in patients with metastatic melanoma

P. Hersey[1]*, E. Hasic[1], M. MacDonald[1], A. Edwards[1], A. Spurling[1], A.S. Coates[2,3], G.W. Milton[2] & W.H. McCarthy[2]

[1]*Immunology Unit, Medical Research Department, Kanematsu Memorial Institute, Sydney Hospital, Sydney, N.S.W. 2000;* [2]*The Sydney Melanoma Unit, Royal Prince Alfred Hospital, Camperdown, N.S.W. 2050; and* [3]*Ludwig Institute for Cancer Research, Sydney Branch, University of Sydney, Sydney, N.S.W. 2006 Australia*

**Summary** Studies were initiated to assess the response of patients with disseminated melanoma to recombinant α interferon (rIFN-αA) and to monitor effects of rIFN-αA on several tests of immune function. Twenty patients were treated with rIFN-αA given by i.m. injection in escalating doses from 15 to $50 \times 10^6 \mathrm{u\,m^{-2}}$. The responses of two patients were considered unevaluable. Of the remainder there was complete remission of tumour in two and stable disease in two. Subsequent progression of tumour in one of the latter patients coincided with development of antibodies to IFN. Side effects (usually fatigue) were dose rate limiting in 11 patients.

Laboratory tests on samples taken 6 hours after rIFN-αA indicated a marked lymphopenia and a reduction in natural killer (NK) cell activity particularly against K562 target cells. Longer term changes measured in samples taken 2 days after the previous rIFN-αA injections consisted of neutropenia and an increase in the T4/T8 ratio due mainly to a relative increase in OKT4 positive T cells compared to OKT8 positive T cells. NK activity against the K562 target cell increased in most patients during the first week of treatment and then returned to below or near pretreatment levels thereafter against the K562 target cell. This contrasted with NK activity against the melanoma target cell which showed a more gradual increase over the duration of the treatment in 6 patients. The latter correlated with an increase in mitogen stimulated IL 2 production from their blood lymphocytes and may indicate that the cytotoxic activity resulted from lymphokine-activated killer (LAK) cells.

These results confirm the activity of rIFN-αA against melanoma in certain patients. They suggest that further studies are needed to select patients who may respond to rIFN-αA and to optimize treatment regimens. Tests of IL 2 production and LAK activity may assisted in achieving these objectives.

Systemic therapy for patients with unresectable metastatic melanoma is extremely poor and a variety of reigmens have not altered the median survivals of the disease (Amer *et al.*, 1979; Feun *et al.*, 1982). Recent Phase I and II studies with leukocyte interferon (IFN-α) suggested that IFN-α from whole cells had activity against melanomas in a small proportion of patients (Krown *et al.*, 1981). Similar limited responses were also seen in patients treated with lymphoblastoid IFN (Retsas *et al.*, 1983). The amount of IFN from these sources was limited and it has only been with the advent of recombinant DNA technology that sufficient quantities of purified IFNs have been available for clinical studies over a wide range of dosages and different schedules. Two recombinant IFN-αs have received preliminary evaluation in patients with cancer, rIFN-αA distributed by Hoffman-LaRoche and rIFN-$\alpha_2$ from the Schering-Plough Corporation. rIFN-αA given in similar doses to leukocyte IFN was shown to produce equivalent levels of antiviral activity and to have a similar effects (Gutterman *et al.*, 1982).

In studies on patients with a variety of malignancies, objective responses were mainly seen in patients with lymphoma (Quesada & Gutterman, 1983). Evaluation of these products in patients with melanoma is as yet, at an early stage. In a preliminary study IFN-$\alpha_2$ was given i.v. to 16 patients with disseminated melanoma using various dose regimes. Two patients had complete remissions and 5 had stabilization of their disease (Ernstoff *et al.*, 1983). Similar response rates were reported for rIFN-αA (Creagan, 1983, 1984*a*,*b*).

The purpose of the present study was to further evaluate the therapeutic efficacy of rIFN-αA given in high doses i.m. in patients with metastatic

*Present address: P. Hersey, Department of Oncology & Immunology, 4th Floor, David Maddison Clinical Sciences Building, Royal Newcastle Hospital, Newcastle. N.S.W. 2300 Australia.

Received 12 June 1984; and in revised form 8 February 1985.

© The Macmillan Press Ltd., 1985

melanoma and to monitor a variety of immune responses to determine whether these may assist in optimizing treatment in subsequent studies.

### Materials and methods

### *Patients studied*

These were 15 patients attending the Sydney Melanoma Unit and 5 patients attending the Newcastle Melanoma Unit, with metastases from melanoma. A summary of their clinical details is given in Table I. Twelve were in good physical condition with a performance status of 0–1 assessed on a 5 point scale (Miller *et al.*, 1981) whereas 8 had restriction on activities due to their disease. Eleven had no previous treatment and two had received vaccinations with a melanoma cell vaccinia lysate. Only 5 patients (2, 3, 13, 16 and 19) had chemotherapy and/or radiotherapy within the preceding 6 months.

### *IFN administration and study design*

rIFN-$\alpha$A (RO 22-8181) was supplied by Roche products (Dee Why, N.S.W.) as a freeze dried preparation in ampoules of 3, 18 and $50 \times 10^6$ u ml$^{-1}$ (Mu ml$^{-1}$). The preparation was reconstituted immediately before use and given by i.m. injection in the gluteal region 3 times a week on a Monday, Wednesday and Friday.

All patients had thorough clinical examinations and careful staging of their disease including computerized tomography (when appropriate) before initiation of rIFN-$\alpha$A treatment. Complete blood cell counts, differential count and platelet counts, blood chemistry, urinalysis, electrocardiogram and chest X-ray were performed prior to commencement of treatment. Patients were admitted to hospital for initiation of treatment and rIFN-$\alpha$ given in escalating doses of 15 and 30 Mu m$^{-2}$ on two separate occasions and then 50 Mu m$^{-2}$ thereafter.

Vital signs were recorded before each injection and hourly for 4–6 h post injection. All patients were followed up by physical examination at two weekly intervals. Peripheral blood cell counts were repeated prior to each injection, in the first week then each week for 6 weeks and each 2 weeks thereafter. Blood chemistry was repeated once a week for 6 weeks then each 4 weeks. Chest X-ray and urinalysis was repeated each 4 weeks. Tumour status was reassessed at least once a month by tests pertinent to each patient. Treatment was discontinued if severe toxicity (Miller *et al.*, 1981) and/or tumour progression became apparent.

### *Criteria of response*

Following the recommendations of Miller *et al.* (1981), complete response was defined as the disappearance of all clinical evidence of active tumour for a minimum of 4 weeks. Partial response was defined as a 50% or greater decrease in the products of the perpendicular diameters of measurable lesions with no progression of any existing lesions or the development of new lesions. Stable disease was defined as no response and $<25\%$ increase in the size of lesions and the absence of new lesions. Progressive disease was defined as an increase of $>25\%$ in the size of the measurable lesions or the appearance of new lesions.

### *Immunological tests*

*Estimation of lymphocytes subpopulations in blood* Mononuclear cells were separated from defibrinated blood samples by centrifugation on hypaque-ficoll using standard techniques. T cell populations were defined by use of monoclonal antibodies OKT3, OKT4 and OKT8 (Ortho Diagnostics, North Ryde, N.S.W.) and peroxidase labelled rabbit anti-mouse immunoglobulin (Dako Patts, Code, P. 161).

Cells ($3$–$5 \times 10^5$) in 30 $\mu$l were placed on a slide, air dried and fixed in acetone for 5 min. They were washed in PBS, incubated with 0.03% $H_2O_2$ for 5 min and washed with PBS + 1%BSA. Twenty microlitres of a 1 in 4 dilution of the monoclonal antibody was added for 30 min at room temperature and after washing the cells were incubated in a 1:20 dilution of the second antibody. They were washed and the incubated with the substrate, 3-amino-9-ethyl-ethylcarbazole, prepared as described elsewhere, (Hersey *et al.*, 1983) for 15 min, stained with haematoxylin and mounted in Glycergel (Dako) for examination by bright field microscopy.

### *Measurement of NK activity*

The methods used to measure NK activity against the MM200 and K562 myeloid cells in $^{51}$Cr release assays are described elsewhere (Hersey *et al.*, 1980, 1981). Blood monoculear cells ($3 \times 10^5$, $10^5$, and $3 \times 10^4$ in 0.5 ml) were incubated with $3 \times 10^3$ $^{51}$Cr-labelled MM200 or $10^4$ K562 cells (in 0.5 ml) in overnight 16 h assays in duplicate round bottomed tubes. Supernatants (0.5 ml) were harvested after centrifugation at 500 *g* 7 min and counted. Percentage of $^{51}$Cr release was calculated by the formula $2a/a + b \times 100$, where a = counts in supernatant alone tube minus machine background and b = counts in tube with target cells and half the supernatant. Percent specific cytotoxicity was calculated as follows:

$$\frac{\%\,^{51}\text{Cr release test} - \%\text{ spontaneous }^{51}\text{Cr release}}{\text{Maximum }\%\,^{51}\text{Cr release} - \%\text{ spontaneous }^{51}\text{Cr release}} \times 100$$

Lytic units were defined as the number of effector cells required to lyse 20% of the target cells and were expressed per $10^6$ of the lymphocyte population [LU(20%/$10^6$)] (Pross & Baines, 1982). These values were compared to the mean value of two frozen thawed controls carried out in parallel with the test subjects. If the value of the controls was outside the mean + 2 s.e. of the mean control value (estimated form ≧20 tests), the test value was multiplied by a multiple obtained by dividing the control value on that day by the mean control value.

*Measurement of IL2 production and assay of IL2*

Blood lymphocytes, $4 \times 10^6$ in 2 ml of RPMI (no FCS), were incubated with 1% PHA (Wellcome Pharmaceuticals, Code HA15) for 36 h in flat bottomed Bijou bottles. The supernatants were collected and assayed at 4 dilutions for mitogenic activity against the NK-7, IL 2 dependent, murine cell line (111-E3) kindly provided by Professor Kumagai and described elsewhere (Suzuki *et al.*,, 1983). In brief, supernatant (100 μl) was added in doubling dilutions to triplicate cells with $2 \times 10^4$ NK-7 cells for 18 h and pulsed with 1 μCi $^{125}$IUDR for 6 h. A unit of IL2 was defined as the reciprocal dilution that induced 50% of the maximum IUDR uptake of a standard IL 2 preparation included in each assay. To reduce variability, supernatants from IL 2 production assays as above from each patient, were stored until the treatment with interferom was complete and all supernatants assayed at once against the NK-7 cells.

*Statistical analysis*

The complete remission rate and 95% confidence limits were obtained from the binomial distribution tables. Sequential changes in leukocyte populations were tested for significance by analysis of one way variance using data collected on days 1, 3, 10 and 31 using the "minitab" statistical package. Median survival was estimated from life tables constructed as described by Kaplan & Meier (1958). Analysis of the significance of short term changes in NK activity during treatment with rIFN-αA was carried out by paired t test.

### Results

*Effect of rIFN-αA on tumour growth*

With the exception of patients 2 and 10 all patients were considered evaluable. Patient 2 had extensive tumour growth in the cervical region and discomfort from this was increased by the side effects associated with rIFN-αA, leading to cessation of treatment before response could be assessed. Patient 10 had metastatic disease in the neck of the L femur which had been monitored by serial X-rays. After 4 weeks there was an increase in the lytic area seen on X-ray and this was taken as evidence of progression. At operation for insertion of a pin and plate a month later, however, there was no evidence of tumour in tissue removed from the neck of the femur. Residual foci of melanoma cells could not be excluded by this procedure but there was no clinical evidence of recurrent tumour growth at this site to the time of death ~1 year later. (Recurrent spinal metastases were detected 5 months after treatment with rIFN-αA.)

Two patients appeared to have complete remission of tumour growth. Patient 4 had a 1 cm$^2$ nodule in the left lower lung field which resolved completely after 4 weeks of treatment. He reamined well for one year at which time a further s.c. recurrence was detected near the site of his primary. There was no clinical evidence of systemic disease at this time. Patient 20 had 6 s.c. metastases and 4 pulmonary metastases involving both lung fields. These resolved after 10 weeks of treatment. No response was seen in the first 4 weeks of therapy. Patient 8 had extensive liver metastases which was monitored by serial CAT scans and biochemistry. There was no change in the size of the lesions by CAT scans but biochemistry returned towards normal over the 8 month treatment period. On cessation of rIFN-αA there was rapid progression of lesions in the liver by CAT scans and clinical examination and worsening of liver biochemistry. Patient 9 had shown progression of s.c. and pulmonary lesions until commencement of rIFN-αA. These remained stable or decreased slightly in size on treatment for 9 weeks at which time new s.c. nodules and increased size of existing nodules were noted. These events coincided with detection of antibodies to rIFN-αA by radioimmunoassay.

Ten patients had evidence of disease progression within 5 weeks of commencing rIFN-αA treatment and these were patients with poor performance status before initiation of treatment.

*Effects of rIFN-αA on blood leukocyte populations*

Changes in blood leukocyte populations in samples taken from 15 patients treated at the Sydney

**Table I** Clinical details of patients entered into study of response to rIFN-αA

| *Patient no.* | *Performance status (ECOG)* | *Sex* | *Age* | *Primary site* | *Site of metastases* | *Months to detection of metastases* | *rIFN-αA[a] started (months)* | *Previous treatment* | *Duration of treatment (weeks)* | *Outcome* | *Duration of response (weeks)* | *Survival[c] (weeks) (4/1/85)* |
|---|---|---|---|---|---|---|---|---|---|---|---|---|
| 1 | 1 | M | 41 | Trunk | Lung Sacum | 9 | 13 | Nil | 11 | PD | — | 26 |
| 2 | 2 | M | 42 | Head | Cx LN sc Lung | 6 | 9 | Bisantrene DTIC of Radiotherapy | 2 | NE[b] | — | 6 |
| 3 | 2 | F | 61 | Leg | Ing LN sc Abd. LN | 11 | 12 | DTIC | 6 | PD | — | 23 |
| 4 | 0 | M | 68 | Leg | Lung | 48 | 49 | Nil | 8 | CR | 52 | 84A |
| 5 | 1 | F | 44 | Trunk | Sternum sc Trunk | 26 | 30 | Nil | 5 | PD | — | 30 |
| 6 | 1 | M | 48 | Trunk | sc Trunk Lung | 5 | 7 | Imm | 5 | PD | — | 6 |
| 7 | 2 | F | 42 | Leg | Bowel sc Trunk | 73 | 75 | Nil | 5 | PD | — | 6 |
| 8 | 0 | F | 30 | Head | Liver | 34 | 35 | DTIC (1980) | 20 | Stable[b] | 32 | 88 |
| 9 | 0 | M | 53 | Trunk | sc Head Ax LN Lung | 15 | 17 | Imm | 10 | Stable[b] | 9 | 35 |
| 10 | 2 | M | 23 | Trunk | Spine, neck of femur | 8 | 17 | DTIC & Radiotherapy | 4 | NE[b] | — | 50 |
| 11 | 1 | M | 27 | Trunk | sc Trunk | 43 | 47 | Nil | 8 | PD | — | 46 |
| 12 | 2 | M | 29 | Trunk | sc Bowel & Lung | 93 | 95 | Nil | 5 | PD | — | 24 |
| 13 | 1 | F | 43 | Leg | Bowel Lung | 13 11 | 14 | DTIC | 8 | PD | — | 43 |
| 14 | 2 | F | 36 | Trunk | sc Trunk As LN Bowel, Liver | 96 101 104 | 106 | Nil | 4 | PD | — | 35 |
| 15 | 1 | F | 35 | Head | Lung | 18 | 19 | Nil | 8 | PD | — | 14 |
| 16 | 1 | M | 60 | Back | sc Trunk Lung | 39 | 51 | DTIC & BCG Cyclophos. | 4 | PD | — | 30 |
| 17 | 2 | F | 42 | Back | Adominal (ascites) Lung | 96 | 98 | Nil | 4 | PD | — | 4 |
| 18 | 2 | F | 44 | Leg | Abdominal LN (ascites) Lung | 353 | 355 | Nil | 5 | PD | — | 8 |
| 19 | 0 | M | 23 | Occult | sc Head & Neck Lung | 4 | 7 | DTIC & BCG | 8 | PD | — | 22A |
| 20 | 0 | M | 73 | Head | sc Trunk Lung | 8 | 10 | Nil | 20 | CR | 28 | 28A |

A = Alive, PD = Progressive Disease, NE = Not Evaluable, CR = Complete Response, Cx = Cervical, Ax = Axilla, Ing = Inguinal, sc = Subcutaneous, LN = Lymph nodes, Imm = Immunotherapy with vaccinia cell lysates, DTIC = Chemotherapy with dacarbazine.

[a]Months from diagnosis.

[b]See text.

[c]Measured from start of IFN treatment.

Melanoma Unit 6 h after administration of rIFN-αA are summarized in Table II. The total leukocyte count at 6 h post injection was unchanged at the 15 and 30 Mu $m^{-2}$ dose but there was a marked reduction in lymphocyte counts and a corresponding increase in neutrophils. The reduction in lymphocyte count was attributed to a loss of T cells from the circulation which affected both T4 and T8 subpopulations. At the 50 Mu $m^{-2}$ dose there was a reduction in both neutrophils and lymphocytes. (There was a reciprocal increase in B cells at all dose levels.)

In contrast to the short term effects, longer term changes measured in blood samples taken prior to each administration of rIFN-αA, consisted of a reduction in total leukocyte counts which was due mainly to a reduction in neutrophils. Lymphocyte numbers were not markedly reduced and except for patients 3 did not exceed 50% of the pretreatment values. These results are shown in a summarized form in Table III as mean values before and during treatment. Analysis of T cell subsets by monoclonal antibodies revealed that the reduction of the OKT8 population tended to be greater than the T4 population giving an increase in the T4/T8 ratio in 10 of the patients. Exceptions to this were results from studies on patients 4, 6, 8, 12 and 15. These results are summarized in Figure 1 which indicates the change in mean lymphocyte counts whole on treatment in relation to changes in the T4/T8 ratio. The increase in T4/T8 ratio could not be accounted for by changes in lymphocyte counts. There was a significant decrease overall in B cell and monocyte

**Figure 1** Changes in mean T4/T8 ratio in relation to changes in mean lympocyte numbers during treatment with rIFN-αA. Changes in lymphocyte numbers were small and did not appear responsible for the increase in the T4/T8 ratio seen in most patients (10/15).

**Table II** Short term (6 h) effect of rIFN-αA on blood leukocytes[a]

| *Dose Mum*$^{-2}$ | *Total Wbc* (×10 $l^{-1}$) | | *Lym* (×$10^{-9}$ $l^{-1}$) | | *T3%* | | *T4%* | | *T8%* | | *T4/T8* | | *B%* | |
|---|---|---|---|---|---|---|---|---|---|---|---|---|---|---|
| | *am*[b] | *pm* | *am* | *pm* | *am* | *pm* | *am* | *pm* | *am* | *pm* | *am* | *pm* | *am* | *pm* |
| 15($n$=9) | 7.3±1.2 | 7.5±3.5 | 1.34±0.7 | 0.37±0.17 | 73±5 (0.98)[c] | 48±7 (0.18) | 46±6 (0.62) | 31±6 (0.11) | 28±9 (0.38) | 18±9 (0.07) | 1.63±0.8 | 1.57±0.9 | 17±5 | 46±7 |
| 30($n$=7) | 5.2±0.6 | 5.5±2.4 | 1.3±0.5 | 0.35±0.17 | 71±7 (0.92) | 42±13 (0.15) | 48±9 (0.62) | 27±9 (0.09) | 23±4 (0.299) | 15±8 (0.05) | 2.07±0.75 | 1.8±1.8 | 20±6 | 52±18 |
| 50($n$=11) | 4.0±1 | 3.3±0.8 | 1.16±0.4 | 0.55±0.23 | 69±9 (0.80) | 53±9 (0.29) | 46±7 (0.53) | 33±8 (0.18) | 23±7 (0.27) | 20±7 (0.11) | 2.27±0.85 | 2.04±1.52 | 24±12 | 40±10 |

[a]Mean values±1 s.d.

[b]am and pm refer to values immediately before and 6h after rIFN-αA administration.

[c]Values in brackets indicate absolute T cell counts ×$10^{-9}$ $l^{-1}$.

**Figure 2** Sequential changes in NK activity against the K562 target cell. (a) Patients showing an increase in the first week of treatment and (b) those showing a decrease or no change. Mean value$\pm$2 s.e. of the frozen-thawed concurrent control PBL against the K562 target cells was 20.3+4.2 LU/20%/$10^6$ (26 estimations).

**Figure 3** Sequential changes in NK activity against the MM200 target cell showing the different pattern of activity to that seen against the K562 target cell. Increased NK activity was seen in most of the patients and this tended to be gradual and prolonged compared to that against the K562 target cells. Mean value$\pm$2 s.e. of the frozen thawed concurrent control PBL against the MM200 target cells was 9.8+3.1LU/20%/$10^6$ (26 estimations). S refers to date of cessation of rIFN-αA treatment.

**Table III** Long term effects on blood leukocytes measured in blood samples taken prior to administration of rIFNαA[a]

| | *Total Wbc* | *Lymphocytes* | *T3* | *T4* | *T8* | *T4/T8* | *B cells* | *Mono* | *Platelets* |
|---|---|---|---|---|---|---|---|---|---|
| Pretreatment | 7.3±1.2 | 1.4±0.6 | 1.0±0.45 | 0.6±0.3 | 0.35±0.2 | 1.9±0.8 | 0.30±0.17 | 0.38±0.2 | 326±82 |
| Day 3 | 5.2±1.4 | 1.5±0.6 | 1.1±0.4 | 0.7±0.2 | 0.37±0.2 | 2.1±0.7 | 0.33±0.20 | 0.36±0.16 | 277±75 |
| Day 10 | 3.8±1.1 | 1.2±0.4 | 0.8±0.3 | 0.5±0.2 | 0.27±0.1 | 2.2±0.9 | 0.27±0.09 | 0.22±0.1 | 247±55 |
| Day 30 | 3.6±1.0 | 1.0±0.4 | 0.7±0.4 | 0.4±0.3 | 0.21±0.1 | 2.0±0.7 | 0.14±0.7 | 0.21±0.1 | 222±63 |
| F value | 23.4 | 2.15 | 1.99 | 2.12 | 1.93 | 0.27 | 3.76 | 3.3 | 4.58 |
| *P* | <0.01 | NS | NS | NS | NS | NS | <0.05 | <0.05 | <0.01 |

[a]Values indicated are mean counts±s.d. ($\times 10^{-9} l^{-1}$). T4/T8 is the ratio of these 2 counts.

**Table IV** Short term (6 h) effects of rIFN-αA on NK activity[a]

| *rIFN-αA* | *K562* | | | *MM200* | | |
|---|---|---|---|---|---|---|
| | *am*[b] | *pm* | *P*[c] | *am* | *pm* | *P* |
| 15 (*n*=10) | 30.7±11 | 6 ±4.6 | <0.001 | 16.4±16.5 | 20.2±34 | NS |
| 30 (*n*=7) | 85±72 | 19±13 | <0.05 | 21.3±14 | 25.5±19.7 | NS |
| 50 (*n*=8) | 44.6±35.8 | 18.8±15.7 | >0.10 | 23±17.8 | 24.5±25 | NS |

[a]LU/20%/10[6] lymphocytes mean±s.d.
[b]am and pm refer to values before and 6 h after rIFN-αA administration.
[c]*P* values determined by paired t test (t was 6.47, 2.46 and 1.72 respectively).

numbers. Platelet counts showed a decrease in all patients (except patient 2) and returned to pretreatment values on cessation of rIFN-αA.

### *Changes in NK activity after rIFN-αA administration*

NK activity in lytic units against the K562 and MM200 target cells. in blood samples taken before and 6 h after i.m. injection of IFN is summarized in Table IV. NK activity against the K562 target cells was markedly reduced in all instances. This was particularly evident at the lower doses of rIFN-αA. In contrast changes in the NK activity at 6h post injection against the MM200 target cell were unchaged or tended to increase.

Longer term changes in NK activity measured in blood samples taken prior to each injection of rIFN-αA (and ~48 h after the last injection) are indicated for all patients except No. 12 in Figures 2 and 3. After initiation of treatment (in tests on day 3 or 5), NK activity against the K562 target cell increased in 9 patients and either decreased (patient no. 3, 7 and 8) or was essentially unchanged (patient nos. 14 and 15) (Figures 2a and 2b respectively.) NK activity returned to pretreatment or lower levels at day 7 or 9 of treatment and increases above pretreatment levels were seen only in patients 1, 4, 7, 8 and 9 after this period. In contrast NK activity increased in lymphocytes from 10 of the 14 patients against the MM200 target cells (Figure 3). Exceptions were patients 1, 3, 8 and 11. The increase was most prominent in those with low pretreatment values and tended to be more gradual than that noted against the K562 target cell. Increases in NK activity MM200 but not K562 were seen in studies on patients 4, 5, 6, 14 and 15.

### *Effects of rIFN-αA on IL 2 production*

IL 2 production by blood lymphocytes taken at intervals during rIFN-αA administration is shown in Figure 4. In 12 patients (No. 14 excepted) IL 2 production was less in samples taken during the first week of IFN treatment but recovered to or above pretreatment levels in the second or subsequent week or treatment. In 6 patients the changes in IL 2 production after the first week of treatment tended to parallel the changes in lymphocyte cytotoxicity against the MM200 target cell e.g. in studies on patient No. 1, 3, 6, 14 and 15. IL 2 production did not correlate with changes in the T4 or T8 T cell populations.

**Figure 4** Sequential changes in IL 2 production from mitogen (PHA) stimulated PBL during treatment with rIFN-αA. In practically all patients there was an initial decrease in IL 2 production at 3rd day of treatment followed by a return to or above pretreatment levels. Mean ± 1 s.d. of IL 2 production from 19 normal concurrent controls was 9.97 + 2.94 units.

### Discussion

The response rate of melanoma to treatment by rIFN-αA in this study consisted of two complete remissions and stabilization of disease in two patients. One patient undergoing complete remission had a solitary pulmonary nodule and the other both pulmonary and s.c. nodules. Previous studies have also reported that responses to IFN-α were confined to metastases in skin (Retsas *et al.*, 1983) or to skin and pulmonary regions (Creagan *et al.*, 1984*a,b;* Pouillart *et al.*, 1984). It was noticeable that responses were confined to patients with a good performance status and there were no responses in those with restricted physical activity due to their disease. This contrasted with the study of Creagan *et al.*, (1984*b*) who observed responses in both good and poor risk patients. Only one patient with extensive disease showed any response. This was a patient with extensive hepatic metastases which remained stable during treatment for a period of 8 months.

Evaluation of the patient with a bony metastases in the neck of the femur was difficult in that there appeared to be disease progression upon X-ray but there was no evidence of melanoma in scrapings obtained at operation for pin and plate of the hip. There was no evidence of tumour growth at this site over approximately a year so that this patient may have had a partial or complete response at this site. If this patient is included in the evaluation and the patient who only had 2 weeks of treatment is excluded the complete response rate was 3/19 patients or 16% (95% confidence levels 3.4–39.6%). If the patient is considered as unevaluable the complete response rate was 2/18 or 11% (95% confidence levels 1.4–34.77%). Median survival for all study participants from the beginning of treatment was 26 weeks by Kaplan-Meier life table analysis (Patient numbers are considered insufficient to make comparisons with published survivals of disseminated melanoma which is ~6 months overall (Balch *et al.*, 1985).

As reported in other studies (Quesada & Gutterman, 1983) rIFN-αA administration was accompanied by a number of side effects which were severe enough to require dosage reduction from the 50 Mu m$^{-2}$ dose in 11 of the 15 patients (see Appendix 1). This was usually from fatigue or a combination of fatigue and nausea. Severe hair loss was noticed in one patient after six months of treatment and grade three liver toxicity (Miller *et al.*, 1981) was detected in two patients. Most patients were able to tolerate the 30 Mu m$^{-2}$ dose and this dose level or less would appear more realistic in future studies.

One of the objectives of the present study was to monitor effects of rIFN-αA on several haematological and immunological tests. Short term (6 h) effects on leukocyte populations consisted of a marked decrease in lymphocytes and an increase in neutrophils so that the total leukocyte numbers tended to remain unchanged. After the 50 Mu m$^{-2}$ dose neutrophils did not increase, resulting in a decrease in total leukocyte numbers. The drop in lymphocyte numbers appeared to result from a reduction in T cells giving a relative increase in B cells. Lymphocyte numbers had returned towards pretreatment levels by two days prior to the next rIFN-αA injection so that the changes noted at 6 h may have reflected sequestration of T cells from the circulation into sites such as the bone marrow. This is reported to occur after steroid administration (Haynes & Fauci, 1978) and in these patients may have resulted from endogenous steroid production as part of a stress response. The latter may also explain the marked reduction of NK activity in the circulation at 6 h as steroids are known to produce this effect (Clarke *et al.*, 1977; Holbrook *et al.*, 1983). Short term reduction in NK activity after rIFN-αA was also noted in previous studies (Lotzova *et al.*, 1983).

These short term effects on leukocyte populations appeared to be superimposed on a general depression of leukocyte numbers (particularly of neutrophils) and of platelets measured in blood samples taken before the next dose of rIFN-αA. The reduction in lymphocyte numbers was not marked and appeared to affect both B and T cells. The relative sparing of lymphocyte numbers in the blood was also noted in previous studies (Golub *et al.*, 1982) and may indicate selective effects of rIFN-αA on various progenitor cells in the marrow as reported elsewhere (Broxymeyer *et al.*, 1983). Within the T cell population there appeared to be a relative decrease in the T8 population, particularly when total lymphocyte numbers were reduced so that the T4/T8 ratio tended to increase. These changes are similar to those reported by Silver *et al.* (1983) using lymphoblastoid IFN and many reflect growth inhibitory effects of rIFN-αA on T cell regeneration particularly if the T8 population has a higher turnover rate than the T4 population.

Short term changes in NK activity measured at 6 h referred to above, appeared to be superimposed on longer term changes detected in blood samples taken 2 days after rIFN-αA administration. These were characterised, in general, by an increase against both target cells in the first week of administration. NK activity against the K562 target cells then declined to approximate pretreatment levels as described in previous studies using crude leukocyte interferon preparations (Golub *et al.*, 1982). As also noted in previous studies (Lotzova *et al.*, 1983) the increase in NK activity was more marked in patients with low pretreatment values. Although the cytotoxic activity of blood lymphocytes against the two target cells was similar in the first week subsequent rIFN-αA treatment was associated (in 6 patients) with a gradual increase against the MM200 but not the K562 target cell. The latter changes did not correlate with changes in the T4 or T8 populations but did appear to correlate with IL 2 production from mitogen stimulated lymphocytes.

These observations may indicate that the cytotoxic activity against the melanoma cell was due to lymphokine activated killer (LAK) cells. It was shown previously that the incubation of lymphocytes in IL 2 may induce cytotoxic activity against a wide range of target cells including autologous tumour cells (Hersey *et al.*, 1981; Grimm *et al.*, 1982) and are considered similar to the "anomalous killer cells" described by Masucci *et al.*, (1980).

MM200 target cells are relatively resistant to NK activity but are sensitive to killing by LAK cells so that the increase in cytotoxic activity noted against the MM200 target cell but not the K562 cells may indicate endogenous production of LAK cells in response to enhanced IL 2 production. The latter may have been induced by administration of rIFN-αA in the previous studies have shown potentiation of IL 2 production *in vitro* by IFN (Blomgren & Einhorn, 1981).

The relevance of the results from the *in vitro* studies to the *in vivo* effects of rIFN-αA on melanoma growth is at present uncertain. Although NK activity against K562 cells was increased in the first week of treatment this tended to be short lived in most of the patients. Previous studies have also raised doubt concerning the significance of NK activity in control of tumour growth in melanoma patients (Hersey *et al.*, 1984). In view of this and previous studies showing that LAK (but not NK) can kill autologous tumour cells *in vitro* this (IL 2 induced) activity may be more relevant in control of tumour growth. In agreement with this an increase in IL 2 production and cytotoxicity against the MM200 melanoma cells was documented in one of the patients undergoing a complete remission but similar changes were also seen in patients not responding to rIFN-αA. Perhaps in the latter patients other factors such as the size of the tumour burden and release of immunosuppressive factors from tumours may need to be considered. These changes were not observed in the patient with extensive liver metastases who had stabilization of tumour growth over a period of 8 months. Arrest of cell division by IFN as reported elsewhere (Creasey *et al.*, 1980) may have been more important in this patient. Whether other mechanisms of action were responsible for the effects, as reported in experimental studies (Belardelli *et al.*, 1983) is unknown.

With respect to future studies on rIFN-αA the response rate and complications observed in this study would not appear to justify further evaluation of dose regimes above 30 Mu $m^{-2}$. This and other studies do however indicate the agent is effective against melanoma particularly in patients with a good performance status and pulmonary and or subcutaneous metastases. Further studies are required to select such patients who may respond to IFN and to develop optimal treatment regimes. The present studies suggest that measurement of IL 2 production and LAK activity may assist in these aims.

This work was supported in part by funds from the National Health and Medical Research Council, Melanoma Research Fund, Sydney Hospital, Sydney Hospital Foundation for Research and Roche Products. We wish to thank the Haematology Department and Biochemistry Department of the Kanematsu Memorial Institute for assessment of haematological and biochemical changes in the patients. We wish to thank Dr David Kingston of Roche Products Pty. Ltd., for supply of Interferon and nursing Sisters R. Schipper and M. MacJanet for management of the patients and recording of patient data.

**Appendix 1** Side effects experienced during rIFN-αA treatment

| | *Flu-like* | *Fatigue* | *Nausea & vomiting* | *Diarrhoea* | *Hair loss* | *Dose reducton (day)* | *Side effect limiting dose* | *SGPT*[a] | *SGOT* | *LDH* | *GGT* | *Alk. phos.* |
|---|---|---|---|---|---|---|---|---|---|---|---|---|
| B.Mc. | ++ | ++ | + | — | — | Yes (45) | Liver toxicity fatigue | 7 | 7 | 2 | 4 | |
| J.H. | + | +++ | ++ | + | — | No | | | | 6[b] | | |
| J.K. | +++ | +++ | | + | | Yes (21) | Fatigue | | | 3[a] | | |
| F.H. | ++ | +++ | | + | | No | | 2.5 | 2 | | | |
| W.A. | ++ | +++ | ++++ | | | Yes (7, 14, 21) | Fatigue vomiting | — | — | — | — | — |
| K.Fl | + | +++ | | | | Yes (28) | Fatigue | | | 2.6[a] | | |
| K.Fr. | ++ | ++ | +++ | | | Yes (11) | Fatigue, nausea & vomiting | 2 | 4.8[a] | 3.3[a] | 9 | 1.7 |
| J.D. | + | ++ | — | — | ++ | Yes (81) | Fatigue | — | — | 2.7[a] | 5.4[a] | 1.7[a] |
| N.H. | + | ++ | | + | | Yes (28) | Fatigue | 2 | 1.5 | | 4 | |
| B.M. | + | + | | | | No | | 2 | 1.5 | | | |
| R.C. | +++ | +++ | + | — | — | Yes (18) | Fatigue | — | — | — | — | — |
| A.L. | ++ | +++ | | | | Yes (14) | Fatigue | 2 | | 3[a] | | |
| M.P. | + | + | | | | No | | — | — | — | — | — |
| J.P. | +++ | +++ | +++ | | | Yes (15) | Nausea fatigue | — | — | — | — | — |
| R.F. | + | +++ | + | | | Yes (30) | Liver toxicity fatigue | 7 | 7 | 3.0 | 2.5 | |

[a]Values indicated are multiples of normal upper limits for the enzymes.

[b]Disease related increase.

**References**

AMER, M.H., AL-SARRAT, M. & VAITKEVICINS, V.K. (1979). Clinical presentation natural history and prognostic factors in advanced melanoma. *Surg. Gynaecol. Obstet.*, **149,** 168.

BALCH, C.M., SOONG, S.J., SHAW, H.M. & MILTON, G.W. (1985). An analysis of prognostic factors in 4000 patients with cutaneous melanoma. In *Cutaneous Melanoma,* (Eds. Balch & Milton) J.P. Lippincott Co. Philadelphia, p. 346.

BELARDELLI, F., GRESSER, I., MAURY, C., DUVILLARD, P., PRADE, M. & MAUNDURY, M.T. (1983). Antitumor effects of interferon in mice injected with interferon-sensitive and interferon-resistant friend leukaemia cells. III. Inhibition of growth and necrosis of tumors implanted subcutaneously. *Int. J. Cancer,* **31,** 649.

BLOMGREN, H. & EINHORN, S. (1981). Lymphokine production by PHA-stimulated human lymphocytes is enhanced by interferon. *Int. Archs. Allergy and Appl. Immunol.,* **66,** 173.

BROXMEYER, H.E., LU, L., PLATZER, E., FEIT, C., JULIANO, L. & RUBIN, B.Y. (1983). Comparative analysis of the influence of human gamma, alhpa and beta interferons on human multipotential (CFU-G-EMM) erythroid (BFU-E) and granulocyte-macrophage (CFU-GM) progenitor cells. *J. Immunol.,* **131,** 1300.

CLARKE, J.R., GAGNON, R.F., GOTCH, F.M. & 4 others. (1977). The effect of prednisolone on leukocyte function in man – a double blind study. *Clin. Exp. Immunol.,* **28,** 292.

CREAGAN, E.J. (1983). Recombinant leukocyte A interferon (RO 22-8181 rIFN alpha A) in disseminated melanoma. *Proc. Am. Soc. Clin. Oncol.,* **19,** 228.

CREAGAN, E.T., AHMANN, D.L., GREEN, S.J. & 4 others. (1984*a*). Phase II study of recombinant leukocyte A interferon (rIFN-αA) in disseminated malignant melanoma. *Cancer.* (In press.)

CREAGAN, E.T., AHMANN, D.L., GREEN, S.J. & 4 others. (1984*b*). Phase II study of "low dose" recombinant leukocyte A interferon (rIFN-αA) in disseminated malignant melanoma. *Am. J. Clin. Oncol.* (In press.)

CREASEY, A.A., BARTHOLOMEW, J.C. & MERIGAN, J.G. (1980). Role of Go-$G_1$-arrest in the inhibition of tumor cell growth by interferon. *Proc. Natl Acad. Sci.,* **77,** 1471.

ERNSTOFF, M.S., REISS, M. & DAVIS, C.A. (1983). Intravenous recombinant alpha-2 interferon in metastatic melanoma. *Proc. Am. Soc. Clin. Oncol.,* **19,** 222.

FEUN, L.G., M. GUTTERMAN, J., BURGESS, M.A. & 11 others. (1982). The natural history of resectable metastatic melanoma. *Cancer,* **50,** 1656.

GOLUB, S.H., DOREY, F., HARA, D., MORTON, D.L. & BURKE, M.W. (1982). Systemic administration of human leukocyte interferon tö melanoma patients. I. Effects on natural killer function cell populations. *J. Natl Cancer Inst.,* **68,** 703.

GRIMM, E.A., MAZUMDER, A., ZHANG, H.Z. & ROSENBERG, S.A. (1982). Lymphokine activated killer cell phenomenon lysis of natural killer resistant fresh solid tumor cells by interleukin 2-activated autologous human peripheral blood lymphocytes. *J. Exp. Med.,* **155,** 1823.

GUTTERMAN, J.U., FINE, S., QUESADA, J. & 10 others. (1982). Recombinant leukocyte A Interferon: Pharmacokinetics, single-dose tolerance and biologic effects in cancer patients. *Ann. Int. Med.,* **96,** 549.

HAYNES, B.F. & FAUCI, A.S. (1978). The differential effect of *in vivo* hydrocortisone on the kinetics of subpopulations of human peripheral blood thymus-derived lymphocytes. *J. Clin. Invest.,* **61,** 703.

HERSEY, P., EDWARDS, A. & McCARTHY, W.H. (1980). Tumor related changes in natural killer cell activity in melanoma patients. Influence of stage of disease, tumor thickness and age of patient. *Int. J. Cancer,* **25,** 187.

HERSEY, P., BINDON, C., EDWARDS, A., MURRAY, E., PHILLIPS, G. & McCARTHY, W.H. (1981). Induction of cytotoxic activity in human lymphocytes against autologous and allogenic melaboma cells *in vitro* by culture with interleukin 2. *Int. J. Cancer,* **28,** 695.

HERSEY, P., GRACE, J., MURRAY, E., PALMER, A. & McCARTHY, W.H. (1983). Expression of Thy-1 antigen on human melanoma cells. *Int. J. Cancer,* **32,** 21.

HERSEY, P., EDWARDS, A., MILTON, G.W. & McCARTHY, W.H. (1984). No evidence for an association between natural killer cell activity and prognosis in melanoma patients. *Natural Immunity and Cell Growth Regulation.* (In press.)

HOLBROOK, N.J., COX, W.I. & HORNER, H.C. (1983). Direct suppression of natural killer cell activity in human peripheral blood leukocyte cultures by glucocorticoids and its modulation by interferon. *Cancer Res.,* **43,** 4019.

KAPLAN, E.L. & MEIER, P. (1958). Nonparametric estimation for incomplete observations. *J. Am. Statist. Ass.,* **53,** 457.

KROWN, S., BURKE, M., KIRKWOOD, J.M. & 4 others. (1981). Human leukocyte (alpha) interferon in metastatic malignant melanoma. American Cancer Society Phase II Trial. *Cancer Treat. Rep.,* **68,** 723.

LOTZOVA, E., SAVARY, C.A., QUESADA, J.R., GUTTERMAN, J.U. & HERSH, E.M. (1983). Analysis of natural killer cell cytotoxicity of cancer patients treated with recombinant interferon. *J. Natl Cancer Inst.,* **71,** 903.

MASUCCI, M.G., KLEIN, E. & ARGOV, S. (1980). Disappearance of the NK effect after explanation of lymphocytes and generation of similar non-specific cytotoxicity correlated to the level of blastogenesis in activated cultures. *J. Immunol.,* **124,** 2458.

MILLER, A.B., HOOGSTRATEN, B., STAQUET, M. & WINKLER, A. (1981). Reporting results of cancer treatment. *Cancer,* **47,** 207.

POUILLART, P., BRETAUDEAU, B., DORVAL, T. & 5 others. (1984). Clinical phase II study of recombinant DNA interferon (H.U. IFN-α2) in patients with metastatic malignant melanoma. *Antiviral Res.,* **3,** Abst. D19.

PROSS, H.F. & BAINES, M.G. (1980). Studies of human natural killer cells. I. *In vivo* parameters affecting normal cytotoxic function. *Int. J. Cancer,* **19,** 383.

QUESADA, J.R. & GUTTERMAN, J.U. (1983). Clinical studies of recombinant DNA-produced leukocyte interferon (Clone A) in an intermittent schedule in cancer patients. *J. Natl Cancer Inst.*, **70**, 1045.

RETSAS, S., PRIESTMAN, T.J., NEWTON, K.A. & WESTBURY, G. (1983). Evaluation of human lymphoblastoid interferon in advanced malignant melanoma. *Cancer*, **51**, 273.

SILVER, H.K., CONNORS, J.M., KARIM, K.M. & 5 others. (1983). Effect of lymphoblastoid interferon on lymphocyte subsets in cancer patients. *J. Biol. Response Med.*, **2**, 428.

SUZUKI, R., HANDA, K., ITOH, K. & KUMAGAI, K. (1983). Natural killer cells as a responder to interleukin 2 (IL 2). Proliferative response and establishment of cloned cells. *J. Immunol.*, **130**, 981.